# Health literate settings in Austria

**DOI:** 10.1093/eurpub/ckac129.379

**Published:** 2022-10-25

**Authors:** L Gugglberger

**Affiliations:** Austrian National Public Health Institute, Gesundheit Österreich GmbH, Vienna, Austria

## Abstract

**Issue/problem:**

Health literacy (HL) depends largely on how easy or difficult it is in a given situation to find, understand, evaluate and apply health-related information. HL is thus an interplay between personal skills and organisational requirements.

**Description of the problem:**

In 2018 the Austrian platform for HL initiated the focal point ‘organisational framework conditions for strengthening health literacy’. Since then the needs of organisations are being assessed regularly and several strategies and initiatives have been launched to support organisational HL.

**Results:**

To support organisations and settings with the implementation of HL a “starter kit” consisting of a general practical guide and several settings specific self-assessment tools (SAT) has been developed. SAT currently exist for hospitals, primary care units, businesses, open youth work, schools and municipalities. For the settings of primary care units as well as open youth work certification processes have been developed, based on the SAT, distribution of high-quality health information and proof of communication trainings. Currently tools for general practitioners are being developed.

**Lessons:**

Experiences from workshops and pilot testing have shown that organisations need a variety of different tools and measures to help with the implementation of HL. Any type of support needs to be flexible and customisable to different needs and have a low threshold. What is needed most - apart from financial incentives - are settings-based workshops and personal consultation to raise awareness of what health literate organisations can achieve.

Main messages: Settings and organisations are important to increase HL in the population. Several settings in Austria have started to implement HL and are at different implementation statuses.

